# Comparative Catalytic Properties of Supported and Encapsulated Gold Nanoparticles in Homocoupling Reactions

**DOI:** 10.3389/fchem.2020.00834

**Published:** 2020-09-15

**Authors:** Wongi Jang, Jaehan Yun, Luke Ludwig, Su Guan Jang, Jae Young Bae, Hongsik Byun, Jun-Hyun Kim

**Affiliations:** ^1^Department of Chemistry, Illinois State University, Normal, IL, United States; ^2^Department of Chemical Engineering, Keimyung University, Daegu, South Korea; ^3^Department of Energy Engineering, Dankook University, Cheonan, South Korea; ^4^Department of Chemistry, Keimyung University, Daegu, South Korea

**Keywords:** gold nanoparticle, deposition precipitation, mesoporous TiO_2_, poly(N-isopropylacrylamide), homocoupling reaction

## Abstract

This report describes strategies to increase the reactive surfaces of integrated gold nanoparticles (AuNPs) by employing two different types of host materials that do not possess strong electrostatic and/or covalent interactive forces. These composite particles are then utilized as highly reactive and recyclable quasi-homogeneous catalysts in a C-C bond forming reaction. The use of mesoporous TiO_2_ and poly(N-isopropylacrylamide), PNIPAM, particles allows for the formation of relatively small and large guest AuNPs and provides the greatly improved stability of the resulting composite particles. As these AuNPs are physically incorporated into the mesoporous TiO_2_ (i.e., supported AuNPs) and PNIPAM particles (i.e., encapsulated AuNPs), their surfaces are maximized to serve as highly reactive catalytic sites. Given their increased physicochemical properties (e.g., stability, dispersity, and surface area), these composite particles exhibit notably high catalytic activity, selectivity, and recyclability in the homocoupling of phenylboronic acid in water and EtOH. Although the small supported AuNPs display slightly faster reaction rates than the large encapsulated AuNPs, the apparent activation energies (E_a_) of both composite particles are comparable, implying no obvious correlation with the size of guest AuNPs under the reaction conditions. Investigating the overall physical properties of various composite particles and their catalytic functions, including the reactivity, selectivity, and E_a_, can lead to the development of highly practical quasi-homogeneous catalysts in green reaction conditions.

## Introduction

In the last few decades, metal-based materials have been extensively fabricated to develop reactive catalysts for various chemical reactions (Astruc et al., 2005; Corma and Garcia, 2008; De Rogatis et al., 2010; Prati and Villa, 2013; Narayan et al., 2019). Nanoscale colloidal metal particles can possibly serve as quasi-homogeneous catalysts to overcome common pitfalls (e.g., recyclability and reactivity) of homogeneous and heterogeneous catalytic systems (Astruc et al., 2005; Prati and Villa, 2013; Price et al., 2019). Among the many metal-based colloidal systems, gold nanoparticles (AuNPs) have shown great potential in fulfilling the need for quasi-homogeneous catalytic properties for several reasons: they have relatively easy-to-control structural features (e.g., size and shape), inherent biocompatible characteristics, and a reasonably high stability when properly modified (Cortie and van der Lingen, 2002; Corma and Garcia, 2008; Piella et al., 2016; Parmentier et al., 2018; Carabineiro, 2019; Tabakova, 2019; Jang et al., 2020). In addition, simple wet chemical synthetic approaches can readily allow for designing structurally diverse colloidal AuNPs possessing these advantages to serve as effective catalysts, even under green reaction conditions. However, many colloidal NPs, including AuNPs in quasi-homogeneous catalytic systems, have shown an intrinsic thermodynamic instability where various strong capping agents are often introduced during synthesis to maintain their stability for many chemical reactions (Han et al., 2009; Fenger et al., 2012; Imura et al., 2016; Rossi et al., 2018). Although the utilization of capping agents allows for the easy control of the structural and physical properties, as well as the catalytic selectivity (Li et al., 2012; Imura et al., 2016; Lu et al., 2018; Rossi et al., 2018), the strong interactive forces between the functional groups of the capping agents and the surfaces of the colloidal NPs generally deter their overall catalytic performance. Specifically, the presence of strong capping agents around the catalytically active sites of AuNPs (e.g., corners, edges, and terraces) greatly diminishes their catalytic functions by blocking the access of reactants during the reactions. This detrimental effect can ideally be avoided if a greatly improved stability of the colloidal AuNPs is achieved in the absence of strong capping agents (Li et al., 2012; Niu and Li, 2014; Imura et al., 2016; May-Masnou et al., 2018; Eyimegwu et al., 2019). Various modification strategies have been explored to prepare AuNPs possessing abundant free surfaces without any capping agents, yet which maintain their stability and catalytic activity.

Here we designed two types of composite particles where AuNPs are physically loaded into mesoporous TiO_2_ (supported AuNPs) and encapsulated into polymer particles (encapsulated AuNPs) *via* very weak interactive forces. Both the supported AuNPs and encapsulated AuNPs exhibited abundant bare surfaces but still displayed great dispersity and stability in water and EtOH. Subsequently, these composite particles were tested as quasi-homogeneous catalysts in homocoupling reactions, which are of particular interest because of their high importance in fundamental chemical production and applicability in the synthesis of bio-active heterocycles, drug-like molecules and natural products (Li and Jin, 2013). In addition, the homocoupling of arylboronic acid in aerobic conditions has been regarded as an effective strategy to form biaryl compounds without using relatively toxic halogen aromatics. Furthermore, these homocoupling reactions sometimes require slightly harsh conditions and a long reaction time to validate the overall catalytic functions of various materials. A strategy to reduce the formation of the phenol byproduct, but to improve biphenyl target product in this reaction, is an additional attractive topic of research that can help to elucidate the selectivity of catalysts (Xu et al., 2017; Chen and Shon, 2018). We have thoroughly compared the reactivity, selectivity, and recyclability of both supported and encapsulated AuNPs in water and EtOH for their possible use as quasi-homogeneous catalysts. The reaction rate and activation energy (E_a_) of these composite particles were also evaluated to understand the effect of capping agent-free surfaces of physically integrated AuNPs during the catalytic reaction. Building upon our previous findings, the comparative study utilizing two different composite particles exhibiting similarities (e.g., bare surface) and discrepancies (e.g., size) could help provide a better understanding of the overall catalytic functions in the homocoupling reactions. In addition, the catalytic activity and overall reaction conditions of these composite particles in the homocoupling of phenylboronic acid are thoroughly compared to reported systems in literature (Supplementary Table S1). As such, investigating these types of composite colloidal materials in chemical reactions can allow for the development of high-yielding, cost-effective, and green quasi-homogeneous catalytic systems.

## Method

### Preparation of Composite Particles for Catalytic Homocoupling Reactions

#### Integration of Gold Nanoparticles Into Mesoporous TiO_2_ Particles (Supported AuNPs)

Mesoporous TiO_2_ particles were prepared by a modification of the sol-gel method (Yoo et al., 2005; Niu et al., 2018). An aliquot (20 mL) of titanium tetraisopropoxide (TTIP) was diluted with 200 mL water. Concentrated HCl (30 mL) was then added to this mixture, which was kept at 90°C. After stirring for 24 h, 60 mL of P123 (10 wt% in water) and 3 mL of mesitylene were added to the resulting mixture, which was cooled to room temperature by stirring for additional 1 h. Finally, 40 mL of NH_4_OH was added and stirred at room temperature for 24 h. The resulting nanoparticles were centrifuged and washed with water, followed by drying at 80°C overnight. The powder was uniformly ground in a mortar and then calcined at 500°C for 5 h using the ramping temperature of 3°C/min.

The loading of AuNPs onto TiO_2_ particles was accomplished by a slight modification of the deposition precipitation method (Zanella et al., 2005; Ma et al., 2008). Specifically, 5.0 mL of 10 mM HAuCl_4_ (0.0394 g/10 mL water) was mixed with 0.4 mL of 1 M KOH in a glass vial (~10.5 pH). This mixture was stirred for 5 min, followed by heating to 80°C using an oil bath. TiO_2_ particles (0.09 g) were then introduced to the heated mixture, which was stirred for additional 2 h. After cooling the mixture to room temperature, it was centrifuged at 6,000 rpm for 30 min three times to remove the free gold ions. The final precipitates were dried at 50°C overnight, resulting in gray powders. The powders were thermally treated in an oven at 210°C for 2 h prior to use as catalysts. The integration of AuNPs into nonporous TiO_2_ particles was accomplished using the same process.

#### Integration of Gold Nanoparticles Into Polymer Particles (Encapsulated AuNPs)

**Scheme 1:**
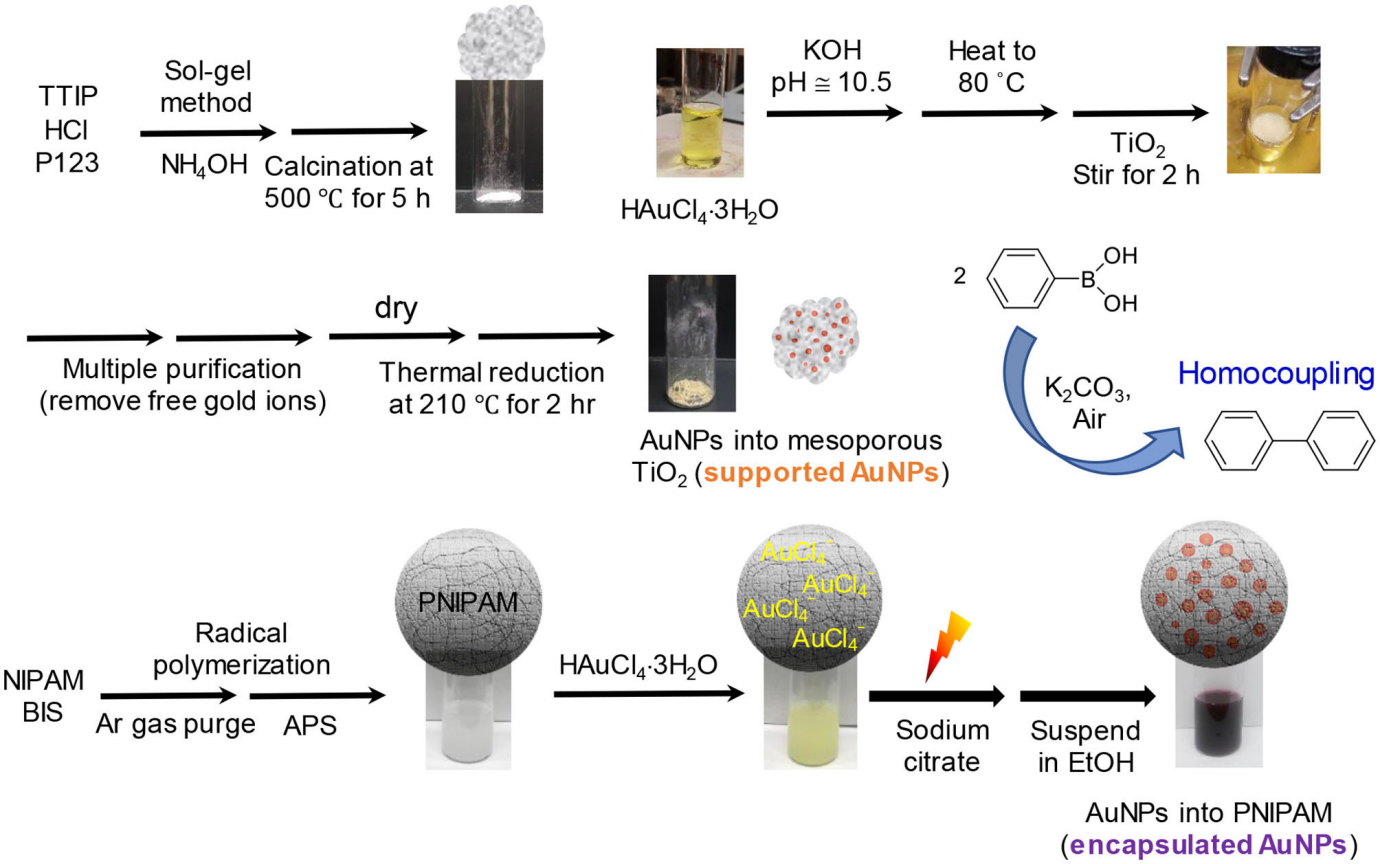
Overall process to prepare the supported and encapsulated AuNPs as reactive catalysts for homocoupling applications.

Poly(N-isopropylacrylamide), PNIPAM, particles were initially synthesized in water *via* radical polymerization (Bergbreiter et al., 1998; Jang et al., 2019). The subsequent loading of AuNPs into the PNIPAM particles was also achieved in water by a light-induced reduction method (Eyimegwu and Kim, 2019; Eyimegwu et al., 2019). Specifically, an aliquot of a PNIPAM solution (10 mL) was mixed with 1.0 mL of 1 wt% HAuCl_4_·3H_2_O for 30 min in a water-jacketed beaker. A trisodium citrate solution (1.0 mL of 1.0 wt%) was added to the reaction mixture, which was placed under a desk lamp (~85 mW/cm^2^) for 3 h. The reaction was fully completed after stirring a minimum of 5 h without the light source. The formation of AuNPs was easily observed by the solution's color change from light yellow to red. The encapsulated AuNPs were then purified in water or EtOH by centrifugation (6,000 rpm for 20 min × 3) to remove the free AuNPs and unreacted species. The overall preparation processes for the supported and encapsulated AuNPs and their catalytic reactions are summarized below (Scheme 1).

### Homocoupling Reactions

The homocoupling of phenylboronic acid was performed in water or EtOH using the supported AuNPs and encapsulated AuNPs. Specifically, an aliquot of the purified composite particles (10 mg for supported AuNPs or 2.0 mL for encapsulated AuNPs) was mixed with phenylboronic acid (21 mg, 0.17 mmol) and K_2_CO_3_ (67 mg, 0.48 mmol) in a glass vial. After a brief sonication, the reaction proceeded under stirring at various temperatures as a function of time. An aliquot of the reaction mixture was then transferred to an Eppendorf tube and separated by centrifugation at 10,000 rpm for 5 min. For the reaction in EtOH, the top EtOH layer (1.0 mL) was directly subjected to GC analysis. For the reaction in water, the water layer was gently extracted with 2.0 mL of diethyl ether. The ether layer (1.0 mL) was then subjected to the GC analysis. A small amount of octane (5 μL) was used as an internal standard for all samples. The recyclability of the composite nanoparticles was tested upon recovering the precipitated composite particles by centrifugation at 6,000 rpm for 20 min twice. Bare AuNPs and nonporous TiO_2_ particles loaded with AuNPs were also tested under the same reaction conditions but underwent severe aggregations (black precipitation or red chunk formation, respectively) in 30 min, indicating a poor stability and dispersity throughout the reactions.

## Results and Discussion

**Figure 1:**
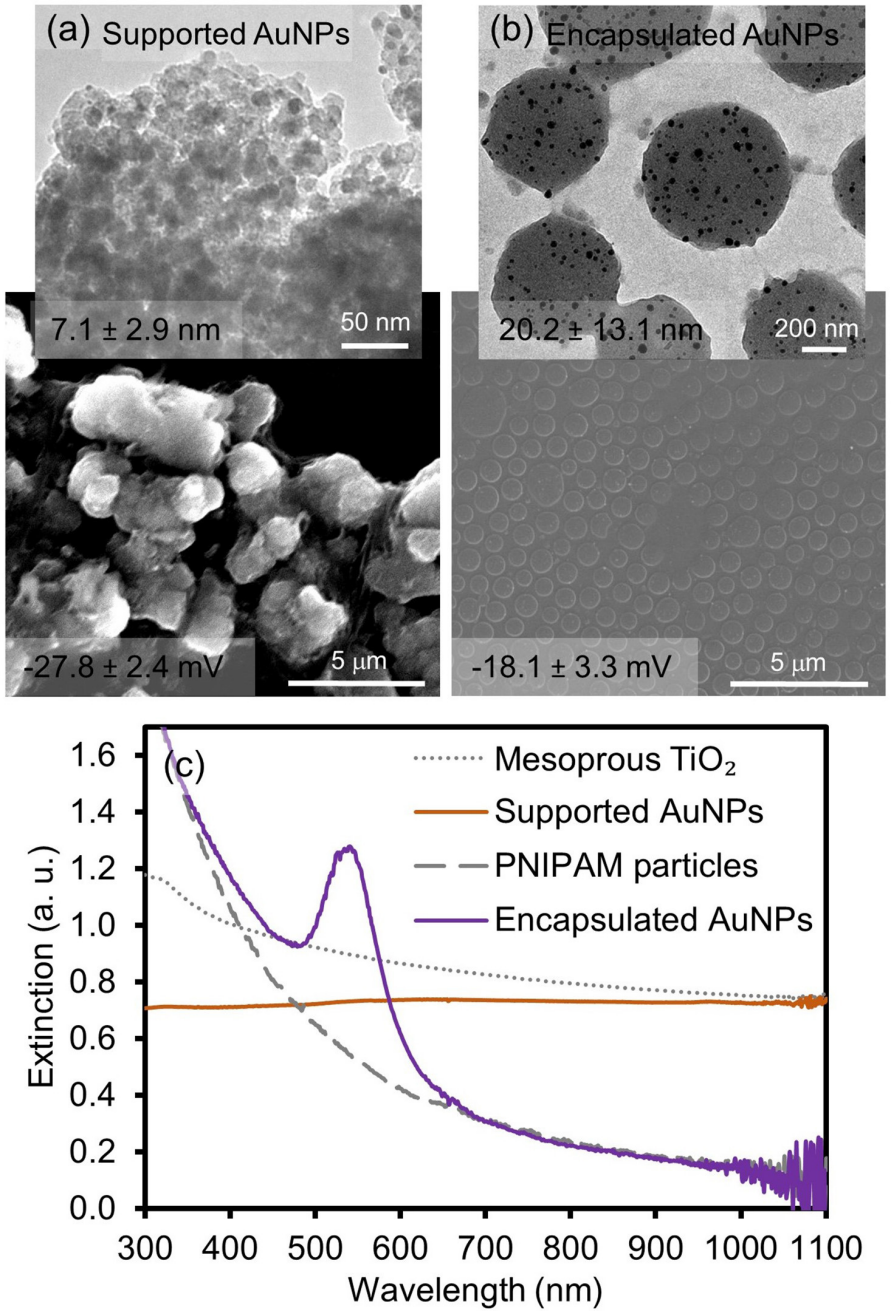
SEM/TEM images of **(a)** supported and **(b)** encapsulated AuNPs and **(c)** their corresponding absorption patterns. The numbers on the images indicate the size of the incorporated AuNPs and the overall surface charge of the composite particles in EtOH.

Figure 1 and Supplementary Figure 1 show the representative images and absorption spectra of supported AuNPs (integrated into the mesoporous TiO_2_ particles) and encapsulated AuNPs (integrated into PNIPAM particles) prepared *via* the deposition precipitation method and light-induced reduction approach, respectively. The surface of the mesoporous TiO_2_ host particles appeared to be somewhat rougher than that of the PNIPAM particles. The pore size of the mesoporous TiO_2_ particles was estimated to be ~9.72 nm (BET test with N_2_ gas shown in Supplementary Figure 1B). A small deviation of the pore size from TEM and BET could be due to the wormhole-like framework structures of the mesoporous TiO_2_ particles. The diameter of the loaded AuNPs into these TiO_2_ particles was analyzed to be 7.1 ± 2.9 nm (TEM). These relatively small and uniform AuNPs were randomly distributed and their surfaces were expected to be free from any modifiers because the synthetic method did not require any reducing and stabilizing agents. On the other hand, the spherical PNIPAM host particles (~590 nm diameter, SEM) encapsulated relatively polydisperse AuNPs (20.2 ± 13.1 nm, TEM), which were formed by the reduction of gold ions in the presence of the trisodium citrate reducing/stabilizing agent. The loading of the AuNPs was then observed by the distinctive surface plasmon resonance (SPR) band at ~538 nm. However, the AuNPs supported onto the TiO_2_ particles displayed a generally flat absorption band across the entire wavelength, except a broad and weak peak at ~550 nm, implying that the dispersity of the composite particles slightly deteriorated upon the integration of the AuNPs. The near absence of a distinctive SPR peak could also be attributed to the poor dispersity of the host TiO_2_ particles (e.g., more light scattering) and the relatively small size of the guest AuNPs (e.g., the weak absorption ability). Our current effort involves the systematic control of the structural features (e.g., size) and loading efficiency for the integrated AuNPs onto the mesoporous TiO_2_ particles.

**Figure 2:**
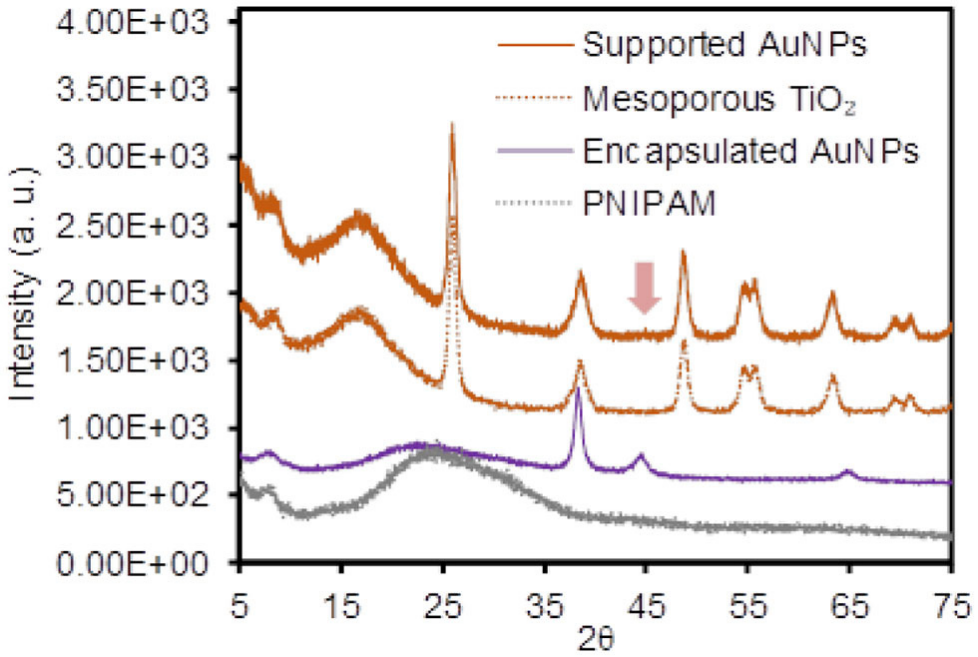
Power X-ray diffraction (PXRD) patterns of the supported and encapsulated AuNPs and their host materials.

To examine the physical integration of the AuNPs (e.g., absence of any modifiers) into the TiO_2_ and PNIPAM host particles, the changes of the surface charges and vibrational peaks were compared before and after the formation of the composite particles by the zeta potential and IR spectroscopy, respectively (Figure 1 and Supplementary Figure 2). Specifically, the negligible changes of the surface charges were observed for the initial TiO_2_ particles (−24.2 ± 5.0 mV) and the supported AuNPs (−27.8 ± 2.4 mV) in EtOH. Similarly, the zeta potentials for the initial PNIPAM particles (−13.4 ± 3.4 mV) and encapsulated AuNPs (−18.1 ± 3.3 mV) were comparable in EtOH. The FT-IR spectra patterns were also identical across the entire vibrational range, which clearly indicated the absence of strong electrostatic or covalent interactions between the guest AuNPs and host materials (i.e., they were free from any stabilizing and/or capping agents). In addition, powder X-ray diffraction (PXRD) spectra were collected to show the presence of supported and encapsulated AuNPs within the mesoporous TiO_2_ and PNIPAM particles (Figure 2). The bare PNIPAM particles showed an amorphous characteristic peak at 2θ = 24.5°, but the encapsulated AuNPs exhibited three distinctive peaks at 2θ = 38.3°, 44.8° and 65.2° for the (111), (200), and (220) planes of the face-centered cubic gold structure with space group Fm3m (Zhang et al., 2018; Eyimegwu et al., 2019). For the mesoporous TiO_2_ particles, the prominent XRD peaks at 2θ = 25.8°, 38.5°, 48.7°, 54.9°, 55.9°, and 63.4° presented the characteristic patterns of mostly anatase phase TiO_2_ (JCPDS card 21-2172) (Nafria et al., 2013; Machin et al., 2017; Solaiyammal and Murugakoothan, 2019). However, the supported AuNPs did not show the detectable characteristic peaks of AuNPs except for a very weak peak at 2θ = 44.8°. This observation could be because the main diffraction peak at 2θ = 38.5° overlaps broadly with the peak of TiO_2_ particles, and the supported AuNPs are too small and/or broadly distributed to generate diffraction peaks, which is also explained by previously reported literature (Nafria et al., 2013; Machin et al., 2017; Solaiyammal and Murugakoothan, 2019). As such, our synthetic methods readily led to the physical adsorption and encapsulation of the AuNPs within the TiO_2_ and PNIPAM particles where these stable supported and encapsulated AuNPs could potentially circumvent the problems associated with the need for capping agents and the reduction of the catalytic performance.

To examine the thermal stability, the thermogravimetric analyzer and differential scanning calorimetry (a dual TGA/DSC system) were used after completely drying the composite particles (Supplementary Figure 3). The supported AuNPs exhibited a negligible weight loss (TGA) and heat flow (DSC), which were expected because the host TiO_2_ particles were calcined to produce a mesoporous structure prior to AuNP loading. As such, the absence of a sharp exothermic peak at 420°C coming from the phase transition from amorphous to anatase clearly supported the mesoporous feature of the TiO_2_ particles (Deorsola and Vallauri, 2008; Khatim et al., 2013; Byun et al., 2017). The encapsulated AuNPs showed a sharp weight loss at 380°C and complete decomposition over 480°C, which corresponded to the host PNIPAM particles. The amount of loaded AuNPs was estimated to be ~6.9 wt% with respect to the PNIPAM particles. To quantitatively determine the amount of integrated AuNPs into both host particles, atomic absorption spectroscopy (AAS) was utilized after treating the composite particles with a mixture of strong acids. Based on the calibration curve obtained from a series of standard solutions (Supplementary Figure 4), the amounts of integrated Au atoms were found to be 0.86 mg of Au per 10 mg of supported AuNP particles (8.6%) and 1.54 mg of Au per 20 mg of encapsulated AuNP particles (7.7%), which were used for the catalytic applications.

**Figure 3:**
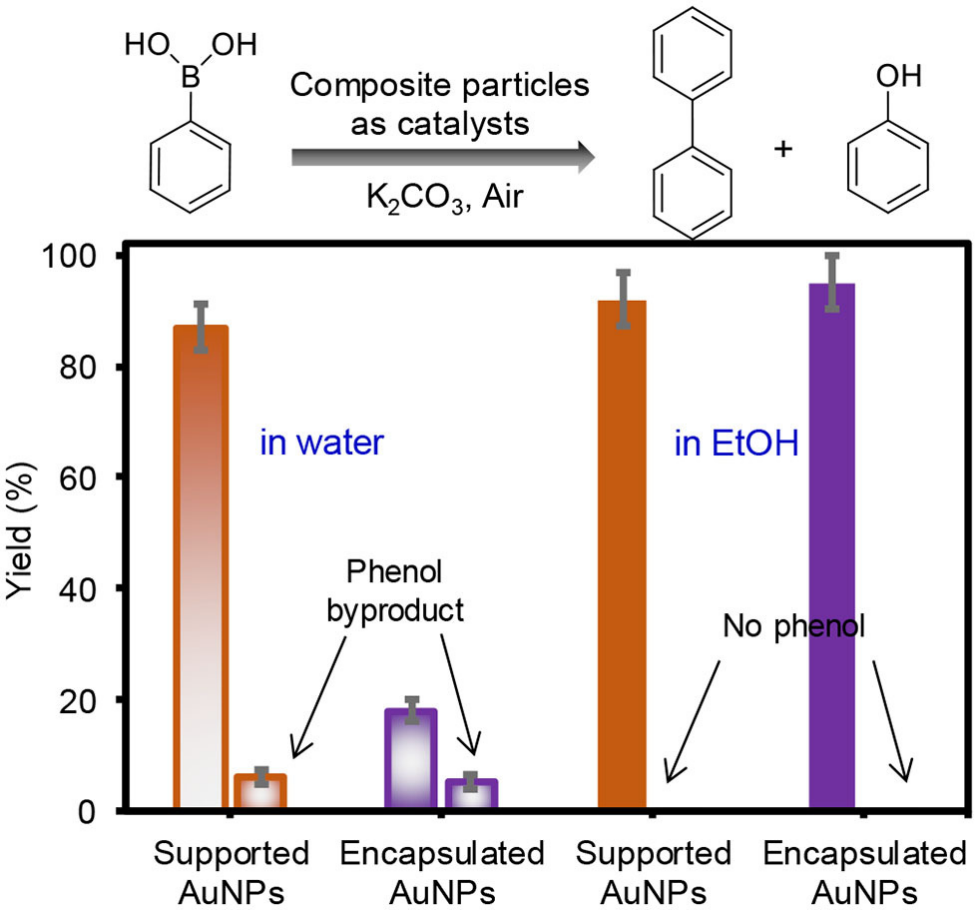
Catalytic yields obtained by GC for the homocoupling reaction of phenylboronic acid using supported and encapsulated AuNPs in water and EtOH (both reactions were performed at 55°C for 4 h under aerobic conditions).

After the characterization of overall physicochemical properties, the supported and encapsulated AuNPs were employed in the homocoupling of phenylboronic acid in water and EtOH under ambient conditions (Figure 3). The composite particles as catalysts resulted in a high-yield reaction, except for the encapsulated AuNPs in water. Specifically, the supported AuNPs showed notably higher reaction yields than the encapsulated AuNPs in water, possibly due to the size of the integrated AuNPs. In addition, the presence of oxidized and/or residual citrate molecules around the surface of the encapsulated AuNPs could block the number of catalytically active sites during the reaction in water, which was also reported in the literature (Li et al., 2012; Niu and Li, 2014). This is because the encapsulated AuNPs formed in the aqueous solution using the trisodium citrate reducing/stabilizing agent exhibited a low zeta potential value (−40 mV), even after extensive purification with water. However, purifying and dispersing the encapsulated AuNPs in EtOH greatly decreased the surface charges (−18 mV), which implied the efficient removal and/or localization of the surface-bound citrate stabilizing agent (Liao et al., 2003; Spina et al., 2017). Reducing the role of the capping agent and maximizing the exposure of catalytic surfaces in EtOH could be one of the reasons for the encapsulated AuNPs to display significantly improved catalytic properties. In addition, the EtOH solvent could improve the mass transfer processes for organic substrates (i.e., phenylboronic acid and biphenyl) during the reaction, which was observed in our recent work (Eyimegwu and Kim, 2019; Eyimegwu et al., 2019). The high yielding reactions using both composite particles in EtOH were clearly observed by FT-NMR spectra which only showed the biphenyl product with near absence of background noise (Supplementary Figure 5). A detectable amount of phenol byproduct was only formed in water, but not in EtOH. This phenol formation in an aqueous system has been explained by the formation of the boron peroxide species in basic reaction conditions (e.g., abundant hydroxide groups and molecular O_2_) (Dhital et al., 2012; Karanjit et al., 2015), and the details of the reaction mechanisms in EtOH are under investigation.

**Figure 4:**
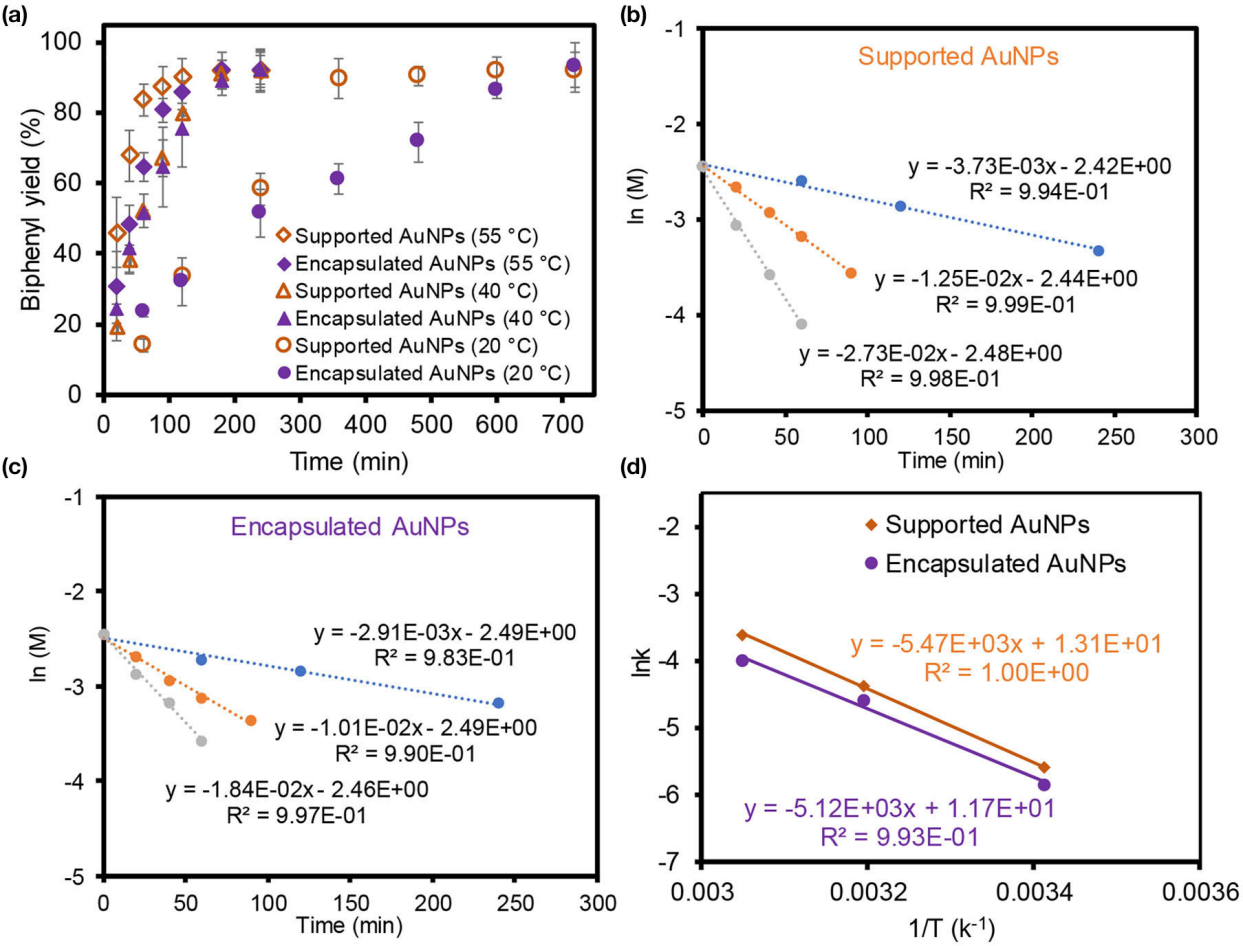
Reaction kinetics **(a)** and Arrhenius plots **(b,c)** to estimated the E_a_ value **(d)** of supported and encapsulated AuNPs in the homocoupling of phenylboronic acid in EtOH.

To further examine the overall catalytic performance of both composite particles, the reaction kinetics and activation energies were obtained after using them in the homocoupling reaction in EtOH (Figure 4). The biphenyl product yields at three different temperatures were examined as a function of time where the supported AuNPs reached the maximum yields slightly faster than the encapsulated AuNPs. Mildly increasing the reaction temperatures rapidly improved the reaction yields for both composite particles. Based on the initial reaction rates, the observed reaction rate constants from the straight slope indicated the first-order reaction for both composite particles. The activation energy (E_a_) was then derived from Arrhenius plots using these three slopes where the apparent E_a_ of the reaction was ~45 kJ/mol for the supported AuNPs and ~43 kJ/mol for the encapsulated AuNPs. The size of both integrated AuNPs was relatively large compared to other reactive AuNP-based catalysts (≤ ~5 nm in diameter), but their abundant bare surfaces that act as catalytically active sites might play an important role to display the comparable E_a_ values within the reported range (27–61 kJ/mol) (Wang et al., 2013; Karanjit et al., 2015; Liu et al., 2016). One can also speculate about the size-dependent E_a_ for the supported and encapsulated AuNPs (Sharma et al., 2003; Fenger et al., 2012; Murzin, 2019), but the comparable E_a_ values, even with notably different sizes and distributions of the AuNPs (e.g., uniform 7 nm vs. polydisperse 20 nm), were possibly due to the compromise effect between the reactivity and dispersity in solution (e.g., higher reactivity but poorer dispersity for supported AuNPs vs. lower reactivity but better dispersity). As the supported AuNPs exhibited relatively high reactivity in water and EtOH, their reaction kinetics were also monitored in water at two different temperatures (Supplementary Figure 6). Using the initial rate of reactions, their apparent E_a_ in water was calculated to be ~44 kJ/mol, which was very comparable to the EtOH solvent system. This observation implied that the catalytic property of the supported AuNPs are not sensitive to these two solvents. More details of the catalytic reactivity and selectivity as well as the reaction kinetics and mechanisms are currently being investigated.

**Figure 5:**
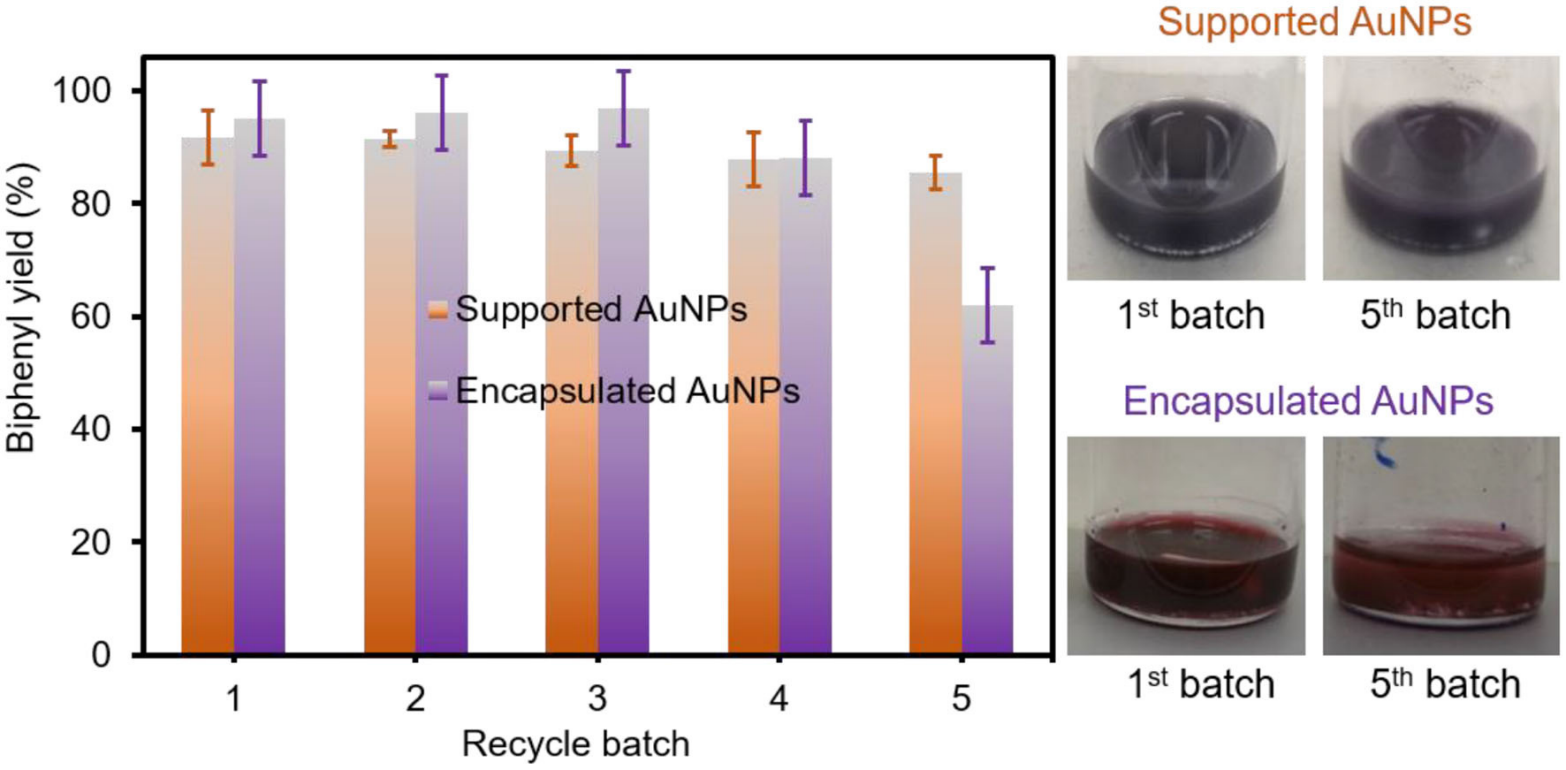
Recycling test of supported and encapsulated AuNPs for the homocoupling reaction in EtOH at ambient conditions (the yield for the dashed bar graph in purple was obtained after washing the encapsulated AuNPs).

The recyclability of both composite particles was then examined in EtOH under aerobic conditions at room temperature (Figure 5). Unlike the catalytic reaction in water, the use of EtOH readily circumvented the extraction step due to the good solubility of the biphenyl product, which also allowed for the easy recovery of the composite particles. Interestingly, the supported AuNPs easily maintained a great catalytic activity at a minimum of six cycles. However, the encapsulated AuNPs were slightly losing their activity due to the obstruction of the host PNIPAM matrix, which was observed in our previous work (Eyimegwu et al., 2019). In contrast to the supported AuNPs, the digital photos also displayed the slight change of the solution color for the encapsulated AuNPs after the fifth batch. Based on the ICP-OES analysis after each recycling test (Supplementary Figure 7), the leaching of AuNPs per cycle was found to be ~0.021 wt% (~0.09 ppm per 430 ppm of total AuNPs) for the supported AuNPs and 0.019 wt% (~0.15 ppm per ~770 ppm of total AuNPs) for the encapsulated AuNPs. Both composite particles exhibited an insignificant loss of AuNPs in each cycle, which could minimally impact the overall catalytic activities. Given the high reactivity and robustness of both composite particles, the homocoupling of arylboronic acid derivatives possessing 4-methyl and 4-methoxy groups was performed at mild temperatures in EtOH (Supplementary Table 2). Both composite particles generally resulted in good reaction yields after 4 h where the slightly lower coupling yield for 4-methoxyboronic acid was obtained due to the limited solubility of the 4,4'-dimethoxybiphenyl product in the EtOH solvent conditions. It is noted that these reactions mainly led to the formation of biaryls with trace levels of phenolic side products. More studies are underway to understand the solubility-related reaction yields under our reaction conditions. These composite particles with controlled physicochemical properties have shown a great potential to serve as reactive, selective, and recyclable catalysts in the homocoupling of phenylboronic acid in EtOH under ambient conditions. Further control of the structural features (e.g., size and shape) and loading amount for integrated AuNPs and understanding their catalytic performance will provide a clear perspective in the development of highly efficient, practical, and green catalytic systems.

## Conclusions

Relatively small and large AuNPs were effectively prepared into mesoporous TiO_2_ (supported AuNPs) and PNIPAM particles (encapsulated AuNPs), respectively, to serve as reactive quasi-homogeneous catalysts. Both kinds of AuNPs were physically incorporated into these host particles in the absence of any stabilizing agents *via* strong electrostatic and covalent bonds, and they exhibited an improved stability in water and EtOH. Upon the utilization of these composite particles in the aerobic homocoupling of phenylboronic acid, the bare surfaces of the integrated AuNPs greatly improved the overall reaction yields by maximizing the interactions between the catalytically active sites and organic reactants. Specifically, the supported AuNPs showed a slightly poor dispersity in water and EtOH but exhibited high catalytic activity and recyclability. The encapsulated AuNPs exhibited good dispersity both in water and EtOH, but showed a high yielding reaction only in EtOH possibly due to the increase of free surfaces and mass transfer processes. Interestingly, these composite particles displayed no clear correlation between the apparent E_a_ values and the size of integrated AuNPs (i.e., size-independent E_a_) in this catalytic reaction. Understanding the way AuNPs are integrated into the host particles and their physiochemical properties in a proper solvent can lead to the significant improvement of their catalytic activity, selectivity, and recyclability.

## Data Availability Statement

All datasets generated for this study are included in the article/Supplementary Material.

## Author Contributions

WJ and JY: They equally conducted the preparation of the encapsulated AuNPs and examined their catalytic properties. They also drafted the initial manuscript. LL: He conducted the preparation of the supported AuNPs and examined the catalytic properties. SJ: He prepared the mesoporous TiO_2_ particles and validated the properties. JB: He supervised the TiO_2_ particle preparation and characterization, and revised the manuscript. HB: He guided the overall catalytic activity tests and revised the manuscript. J-HK: He managed the entire project and arranged the collaborative work. He also finalized the manuscript. All authors contributed to the article and approved the submitted version.

## Conflict of Interest

The authors declare that the research was conducted in the absence of any commercial or financial relationships that could be construed as a potential conflict of interest.
