# Supplementary material for: Comparative Catalytic Properties of Supported and Encapsulated Gold Nanoparticles in Homocoupling Reactions
Source: Front Chem. 2020 Sep 15;8:834. doi: 10.3389/fchem.2020.00834 (PMC7533535; doi:10.3389/fchem.2020.00834)
Supplement: Supplementary file 1 [file Data_Sheet_1.pdf]

Supplementary Material

Enhanced catalytic property of supported and encapsulated gold nanoparticles in homocoupling reactions

Wongi Jang[1,2,3†], Jaehan Yun[1,2†], Luke Ludwig[1], Su Guan Jang,[4] Jae-Young Bae[4]*, Hongsik Byun[2]*, and Jun-Hyun Kim[1]*

[1]Department of Chemistry, Illinois State University, Normal, Illinois 61790-4160
[2]Department of Chemical Engineering, Keimyung University, Daegu 42601, South Korea
[3]Department of Energy Engineering, Dankook University, Cheonan 31116, South Korea
[4]Department of Chemistry, Keimyung University, Daegu 42601, South Korea

[†]These authors contributed equally to this work

*** Correspondence:**
Corresponding Authors
Jae Young Bae (E-mail: jybae@kmu.ac.kr), Hongsik Byun (E-mail: hsbyun@kmu.ac.kr), and Jun-Hyun Kim (E-mail: jkim5@ilstu.edu)

Materials

Ammonium persulfate (APS), N,N'-methylene-bis-acrylamide (BIS), trisodium citrate, potassium carbonate (K_2CO_3), phenylboronic acid, biphenyl, phenol, ethanol, diethyl ether, mesitylene, titanium tetraisopropoxide (TTIP, 97%), ammonium hydroxide (NH_4OH, 25-30 wt% in water), poly(ethylene glycol)-block-poly(propylene glycol)-block-poly(ethylene glycol) (P123), and hydrogen tetrachloroaurate trihydrate ($HAuCl_4{\cdot}3H_2O$) were obtained from Fisher Scientific. 4-Methylphenylboronic acid (98%, Oakwood Chemical), 4-methoxyphenylboronic acid (98%, Acros), 4,4'-dimethylbiphenyl (99%, Alfa Aesar), 4,4'-methoxybiphenyl (>97%, Aldrich) were purchased from the indicated companies. N-isopropylacrylamide (NIPAM, Aldrich) was recrystallized in hexanes and dried under vacuum prior to use. The high purity water used in all reactions was obtained from a Nanopure water system (Barnstead/Thermolyne) with a resistance of 18 MΩ. Glassware was cleaned with an aqua regia solution (a mixture of HCl and HNO_3) or a base bath (a mixture of KOH and isopropyl alcohol), and then rinsed with water.

Characterization

The overall properties of the composite particles and their catalytic properties were evaluated by the combination of scanning electron microscope (SEM), transmission electron microscope (TEM), UV-visible spectrometer (UV-vis), dynamic light scattering (DLS), zeta potential analyzer, Fourier-transform infrared spectroscopy (FTIR), Fourier-transformed nuclear magnetic resonance (FT-NMR), atomic absorption/emission spectroscopy (AA/ICP-OES), a dual thermogravimetric analyzer and differential scanning calorimetry (TGA/DSC) analyzer, powder X-ray diffraction (PXRD) and gas chromatograph (GC).

The diameter distribution and morphology were characterized by SEM (FEI-Quanta 450) and TEM (Hitachi H8100). All samples for the SEM analysis were coated with a thin gold film using a sputter coater (DESKII) after drying them on silicon wafers at room temperature. The samples for the TEM analysis were deposited on 300 mesh carbon-coated copper grids. The size of the embedded and encapsulated AuNPs was estimated from the microscope images using ImageJ software (v1.45s, National Institute of Health) by counting more than 250 nanoparticles. The optical extinction of supported and encapsulated AuNPs were obtained by a UV-visible spectrometer (Agilent) over the wavelength range of 200 to 1100 nm. The purified composite particles were diluted in a quartz cuvette to have the extinction of 1.2. The hydrodynamic diameter/polydispersity of the particle samples and their surface charges were examined with a DLS/zeta potential instrument (ZetaPALS, Brookhaven Instruments Corp.). The particle samples were diluted with water or EtOH (~0.05 mg/mL) for both measurements. The collected data were averaged from a minimum of five measurements. The specific surface area (S_{BET}) of the mesoporous TiO_2 particles (0.2 g) was determined by the adsorption-desorption isotherms of N_2 at -196 °C (QUADRASORB SI apparatus, Quantachrome instrument, Boynton Beach, Florida). All samples were degassed at 200 °C for 3 h under vacuum to remove any adsorbed moisture and impurities. The surface area (S_{BET}) of the sample was calculated by the linear portion of the Brunauer-Emmett-Teller (BET) method based on the N_2 adsorption isotherm results. The total pore volume (V_t) was obtained from the amount adsorbed at a relative pressure (P/P_0) of about 0.99. The pore size distribution was examined by the Barrett-Joyner-Halenda (BJH) method. FTIR (Spectrum 100 FT-IR Spectrometer, PerkinElmer) spectra of TiO_2, PNIPAM, and their composite particles were obtained in the scan range of 4,000 to 650 cm^{-1} using an attenuated total reflection (ATR) sampling device. All samples were fully dried and loaded on an ATR sampling accessory. The integrated amount of AuNPs onto the host materials and the leaching amount of AuNPs during the catalytic reactions were calculated by AA (AAnalyst 200, Perkin Elmer) equipped with an Ag-Au hollow cathode lamp and ICP-OES (Optima 8300, PerkinElmer), respectively. An aliquot of all particle samples (0.1 mL) was treated with a strong acid solution (1.0 mL of a 1:1 volume ratio of HCl to HNO_3) and diluted prior to analysis. The results were compared to those of standard solutions via the Beer−Lambert law. The loading efficiency of AuNPs was also examined by a dual TGA-DSC (SDT Q600, TA Instrument). A small quantity (3 mg) of the particle samples was completely dried in an oven at 50 °C and was placed in an alumina pan for the measurements (pre-heated at 80 ^{o}C for 10 min, ramping temperature of 25 ^{o}C/min from 80 to 800 ^{o}C under N_2 gas). A PXRD system with Cu Kα radiation (MiniFlex 600, Rigaku Corp.) was employed to examine the crystalline features of PNIPAM, mesoporous TiO_2, and composite particles (scan range: 3-80 degree, 0.02 steps, 5 degree/min). All samples were deposited and dried on a glass slide. Gas chromatograms (Thermo Focus GC chromatograph equipped with an FID detector and a fused silica capillary column, Thermo Scientific) were collected to examine the homocoupling reaction yields of the phenylboronic acid. A temperature programming method (a flow rate of 10 psi, a ramping rate of 25 °C/min from 130 °C to 250 °C, and inlet and detector temperatures of 250 °C) was used to isolate each compound peak, which was compared to the calibration curve of the products and byproducts using an octane internal standard. FT-NMR spectra were obtained using a Varian Inova spectrometer 500 and Bruker AVANCE III 500 (499.773 MHz for 1H). Chemical shifts of the 1H NMR spectra were reported in ppm against the residual solvent resonance as the internal standard ($CDCl_3$ = 7.26 ppm).

Supplementary Table 1. Comparison of our composite particles in the catalytic homocoupling of phenylboronic acid over other reported systems in literature.

Sample type	Size of Au (nm)	Reaction conditions	Yield	Recycle	Refs
Au on MAO	1-4	30 mg catalyst (0.7 wt% Au), 100 ℃, O_2, MeOH, 12 h	90	4	Chem. Commun., 2012,48,5476–5478
Au on MAO	1-4	30 mg catalyst (0.7 wt% Au), 100 ℃, O_2, EtOH, 12 h	88	N/A	
Au on MAO	1-4	30 mg catalyst (0.7 wt% Au), 100 ℃, O_2, toluene, 12 h	88.7	N/A	
Au on MAO	1-4	30 mg catalyst (0.7 wt% Au), 100 ℃, O_2, water, 12 h	53.4	N/A	
Au on SiO_2	Not reported	30 mg catalyst (0.7 wt% Au), 100 ℃, O_2, MeOH, 12 h	2.5	N/A	
Au on TiO_2	Not reported	30 mg catalyst (0.7 wt% Au), 100 ℃, O_2, MeOH, 12 h	7	N/A	
Au on C	6	100 mg catalyst (1 wt% Au/C), 45 ℃, air, MeOH, 6 h	25	N/A	ChemCatChem 2018, 10, 1853-1859
Au on MAO-AE	1-4	30 mg catalyst (0.7 wt% Au), 100 ℃, O_2, EtOH, 12 h	88	5	J. Catalysis 2013, 298, 186-197
Au on MAO-HDP	2-8	30 mg catalyst (0.8 wt% Au), 100 ℃, O_2, EtOH, 12 h	92.5	5	
Au on Al_2O_3	Not reported	30 mg catalyst (1.1 wt% Au), 100 ℃, O_2, EtOH, 12 h	12.3	N/A	
Au on ZSM-5	Not reported	30 mg catalyst (0.7 wt% Au), 100 ℃, O_2, EtOH, 12 h	12.6	N/A	
Au on MCM-41	Not reported	30 mg catalyst (1.0 wt% Au), 100 ℃, O_2, EtOH, 12 h	23.9	N/A	
Au-CeO_2	1.3-2.3	1 mol% Au catalyst, room temp., air, water, 7 h	96	5	J. Catalysis 2016, 336, 49-57
Au-CNP	4.9	0.66 mol% Au catalyst, 70 ℃, O_2, toluene-water or water, 7 h	68-86	2	Chem. Commun., 2013, 49, 8235-8237
Au-PVP	1.3	0.5 mol% Au catalyst, 27 ℃, air, K_2CO_3, 24 h	74	N/A	Chem. Commun., 2013, 49, 2542-2544
Au-Chitosan	Not reported	0.5 mol% Au catalyst, 30 ℃, air, acetate buffer, 9 h, additives	93	N/A	
Au-PEG	15	0.02 mol% Au catalyst, 80 ℃, NaOH, water, 48 h	28	N/A	RSC Adv., 2013, 3, 21016
Au-fibrous polymer	Not reported	2 µmol Au catalyst, 70 ℃, K_2CO_3, toluene-water, 7 h	80	N/A	RSC Adv., 2014, 4, 53795
Au-Starch	10	0.625 mol% Au catalyst, K_2CO_3, room temp., water, 48 h	70	5	J. King Saud Univ., Sci., 2017, 29, 547-552
Au-PVP	1.3-1.6	1 atom% Au, K_2CO_3, room temp., water, 24 h	62-72	N/A	Langmuir, 2004, 20, 11293
Au-mesoporous TiO_2	7.1	10 mg catalyst (8.6 % Au), K_2CO_3, 55 ℃, EtOH or water, 4 h	>88	5	Current work
Au-PNIPAM	20.2	20 mg catalyst (7.7 % Au), K_2CO_3, 55 ℃, EtOH, 4 h	>92	5	

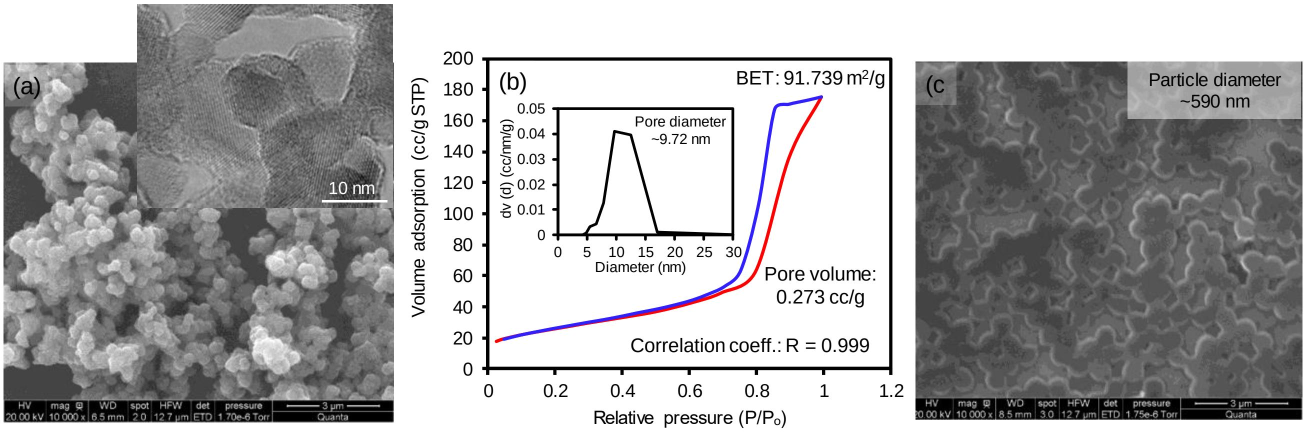

Supplementary Figure 1. The general distribution of host materials: (a) SEM/TEM images and (b) BET of mesoporous TiO_2 particles, and (c) SEM image of PNIPAM particles.

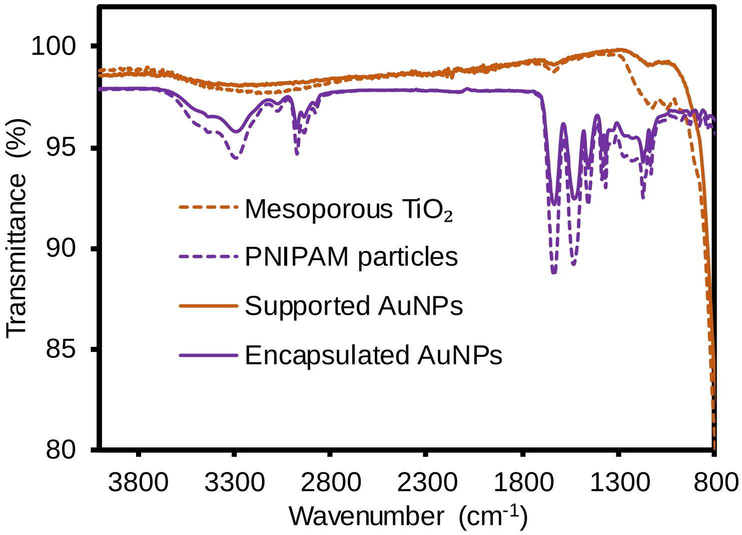

Supplementary Figure 2. FT-IR spectra of the supported and encapsulated AuNPs and their host particles.

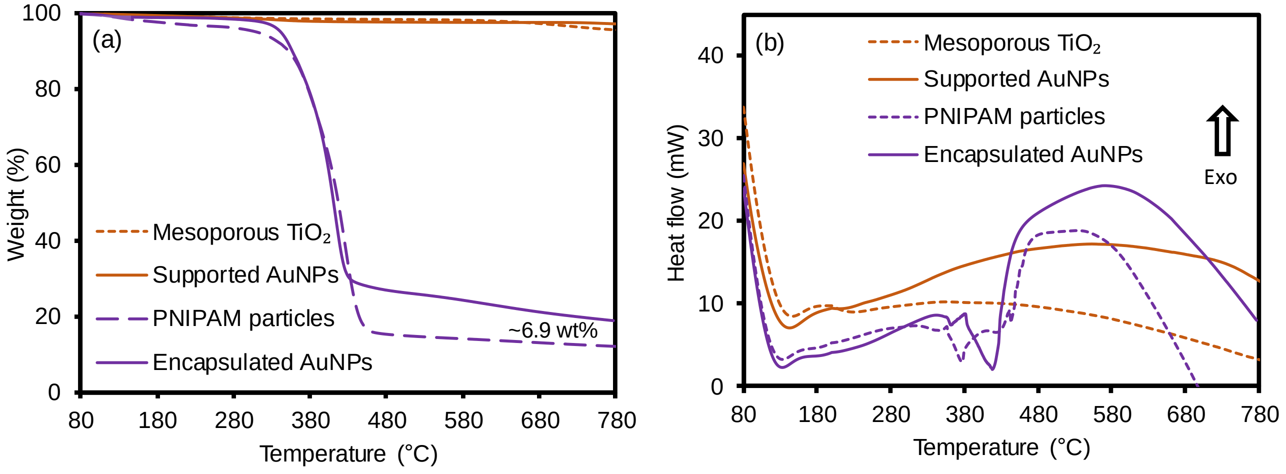

Supplementary Figure 3. (a) Thermogravimetric analysis (TGA) and (b) differential scanning calorimetry (DSC) of the supported and encapsulated AuNPs and their host particles.

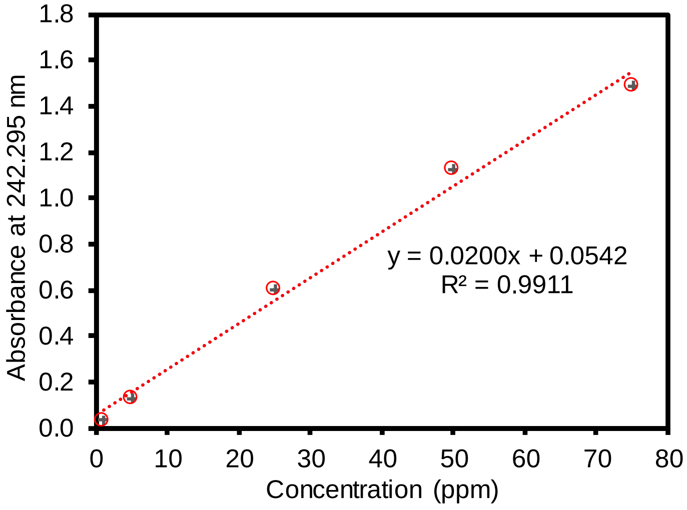

Supplementary Figure 4. A calibration curve for the Au atom obtained by atomic absorption spectroscopy (AAS).

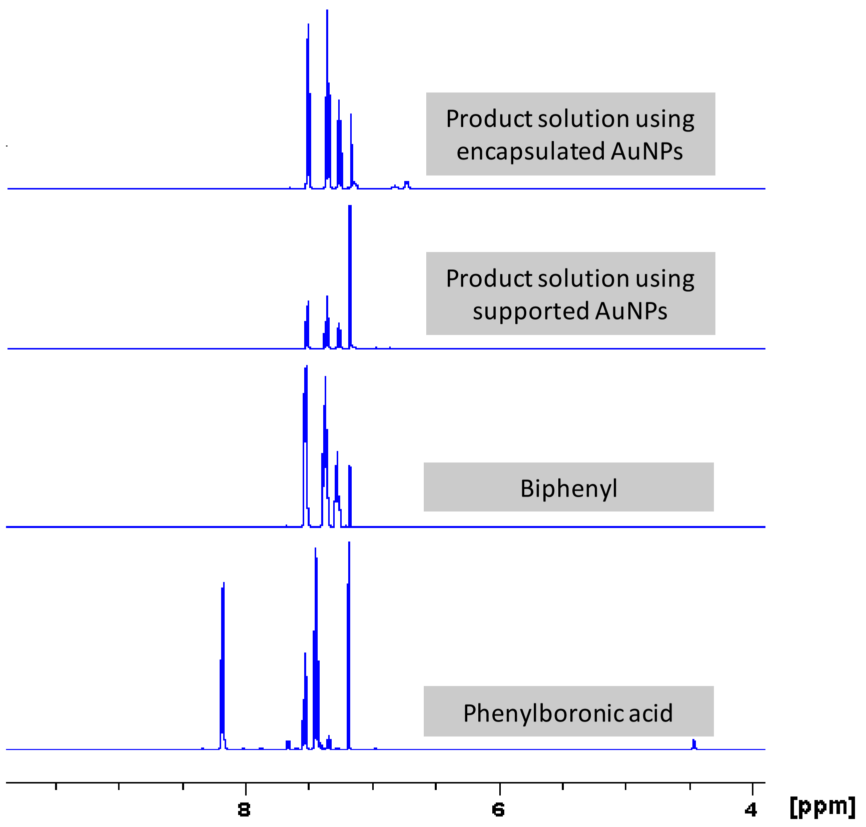

Supplementary Figure 5. FT-NMR spectra of reaction mixture after the homocoupling reaction using the supported and encapsulated AuNPs and the corresponding reactant and product.

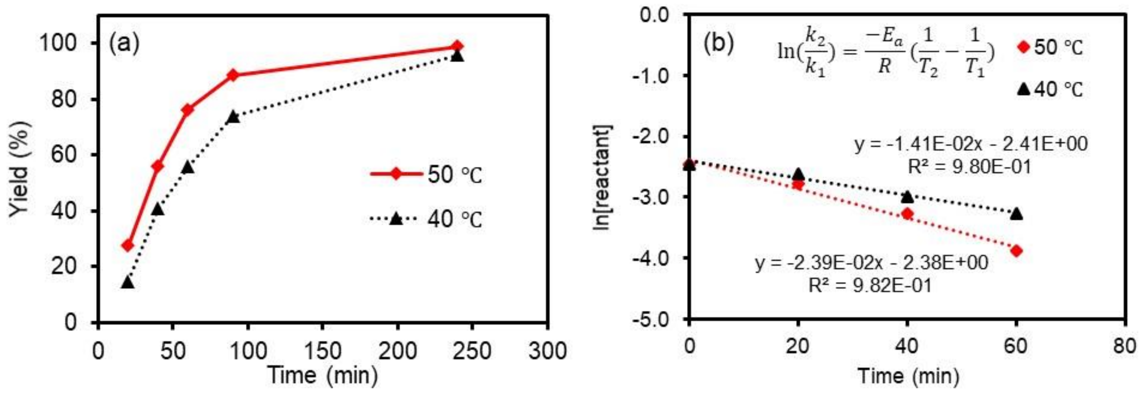

Supplementary Figure 6. (a) Reaction kinetics and (b) Arrhenius plot of the supported AuNPs in the homocoupling of phenylboronic acid in water (the shown equation was used to calculate the E_a value).

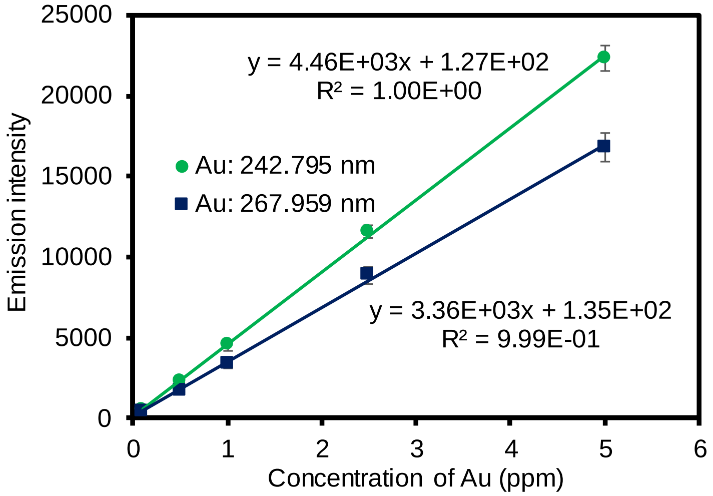

Supplementary Figure 7. Calibration curves for the Au atom obtained by inductively coupled plasma-optical emission spectroscopy (ICP-OES).

Supplementary Table 2. Homocoupling of 4-methylphenylboronic acid and 4-methoxyphenylbornic acid using supported and encapsulated AuNPs in EtOH at 80 ^{o}C for 4 h under aerobic conditions.

Reactant	Product	Yield (%) Supported AuNPs	Yield (%) Encapsulated AuNPs
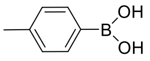	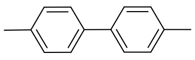	~92 (77)	~96 (85)
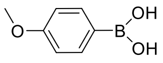	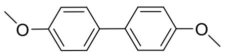	~64 (62)[a)]	~73 (65)[a)]

Reaction at 50 ^{o}C for 4 h under aerobic conditions
[a)]Reaction mixture dried and resuspended in toluene for GC analysis
*Trace levels of byproduct formation (≤ 3%)
Parentheses indicate yields of the same reaction at 55 ^{o}C.
